# Psychosocial and pharmacological interventions for personality disorders in low- and middle-income countries: A systematic review

**DOI:** 10.1371/journal.pgph.0002485

**Published:** 2023-11-03

**Authors:** Thea Lynne Hedemann, North de Pencier, Terri Rodak, Muhammad Ishrat Husain, Usman Arshad, Farooq Naeem, Nasim Chaudhry, Muhammad Omair Husain

**Affiliations:** 1 Department of Psychiatry, University of Toronto, Toronto, Canada; 2 Centre for Addiction and Mental Health, Toronto, Ontario, Canada; 3 Pakistan Institute of Living & Learning, Karachi, Pakistan; 4 University of Manchester, Manchester, United Kingdom; The Valens Clinic, UNITED ARAB EMIRATES

## Abstract

Personality disorders (PDs) have a global prevalence of 7.8% and are associated with increased rates of morbidity and mortality. Most research on PDs has been conducted in High Income Countries (HICs). We conducted a systematic review to investigate the effectiveness of psychosocial and pharmacological interventions for personality disorders (PDs) in individuals from Low- and Middle-Income Countries (LMICs.) We systematically searched MEDLINE, Embase, APA PsycInfo, Web of Science, Cumulative Index of Nursing and Allied Health Literature (CINAHL), and The Cochrane Library from inception to January 5, 2023. Inclusion criteria were quantitative studies and grey literature where participants received a psychosocial or pharmacological intervention for PD. Exclusion criteria were qualitative studies, review articles, studies in which PD was not the primary condition, and articles not available in English. The Cochrane Risk of Bias tool version 2.0 and Joanna Briggs Institute instruments were used to measure risk of bias. Studies were pooled by type of study, PD investigated, type of intervention, assessment methods, and outcomes. Sixteen studies met inclusion criteria and were included. Fifteen were intervention studies related to borderline PD. Only one studied mixed PDs. Twelve studies were of psychotherapy, one pharmacotherapy, one combination of both, and two neurostimulation. Most of the studies showed improvement in symptoms though data was largely collected using self-report measures. There were only six RCTs. There is a dearth of literature on interventions for PDs in LMICs and funding bodies should prioritize research in LMICs. Systematic Review Registration Number: PROSPERO CRD42021233415.

## Introduction

Mental health and substance use disorders are the 7th largest contributors to the global burden of disease. They predominantly impact a younger population with 75% of mental health disorders diagnosed before 24 years of age [1,2]. Young people in low- and middle-income countries (LMICs) are especially vulnerable to developing mental disorders due to high rates of poverty, violence, political instability, trauma, stigma, and humanitarian crises [3,4]. Approximately 80% of individuals suffering from mental health disorders live in LMICs [5]. The high rate of morbidity and mortality associated with mental disorders has prioritized research on effective interventions [6]. Health systems are urged to respond with effective and accessible interventions to reduce lifetime disability and enhance productivity.

Personality disorders are a group of mental health conditions characterized by inflexible and maladaptive patterns of behavior, cognition, and emotion that cause significant distress and impairment in social, occupational, or other areas of functioning [7]. They effect a substantial proportion of the population and have a global prevalence of 7.8% [8]. The exact causes of personality disorders are not fully understood, but genetic and socioeconomic factors have been found to play a role [7]. Personality disorders (PDs) are increasingly recognized as important mental health conditions due to their high morbidity, premature mortality, medical service use, and social costs [9]. Those with PDs have worse outcomes in the key domains of relationships, social functioning, and employment which leads to both individual and societal costs [9,10]. They are also associated with significant distress, functional impairment [11], disability, psychiatric comorbidity, and suicide [9]. Research has shown that PDs can be recognized early in life and respond better to treatment than previously believed [12]. Effective interventions can change functional trajectories and lifelong productivity for many [12].

Effective interventions like psychotherapy have been recommended for individuals with PDs in clinical guidelines published in the United States, United Kingdom, and other high-income countries. Disappointingly, only about 6% of research on mental health disorders emanates from LMICs, and clinicians in LMICs are largely dependent on data from High Income Countries (HICs) to inform care [13]. Interventions in HICs may not be equally feasible or effective in LMICs given social, economic, and cultural differences [14]. With PD interventions often being resource-intensive, it is important to better understand the efficacy of these interventions in LMICs prior to scaling interventions [15].

This systematic review of the literature aimed to identify and summarize effective interventions for individuals with PDs in LMICs. We hope to inform clinicians in LMICs about evidence-based interventions and identify potential gaps in the literature for future research.

## Materials & methods

We conducted the review in accordance with Preferred Reporting Items for Systematic Reviews and Meta-Analysis (PRISMA) guidelines (S1 File) [16]. The protocol was registered with PROSPERO before conducting searches (CRD# 42021233415). The completed review remains aligned with the original PROSPERO protocol in terms of search strategy, research questions and methodology. Any deviations are noted here.

This review aimed to broadly evaluate all research on psychological or pharmacological interventions conducted in LMICs for participants meeting the Diagnostic and Statistical Manual of Mental Disorders (DSM) or International Classification of Disease (ICD) criteria for PD.

### Ethical approval

As this was a systematic review of published studies, ethical approval was not required.

### Search strategy and study selection

A comprehensive search strategy was developed with a health sciences research librarian (TR), which was translated and run in the following bibliographic databases: MEDLINE, Embase, APA PsycInfo, Web of Science, Cumulative Index of Nursing and Allied Health Literature (CINAHL), and The Cochrane Library (see S2 File for full strategies). The search strategies used database-specific subject headings and keywords in natural language to represent two concepts: personality disorders and LMICs. A study type filter was applied, including CADTH’s RCT/CCT search filter and database-specific headings for types of articles to be used for reference list scanning [17]. Publication year limits for all original searches ranged from the date of each database’s inception to January 6, 2021; searches were run again on January 5, 2023 to capture articles published during the review process. No language limits were applied.

These database searches were supplemented by (1) checking for any additional potentially eligible papers cited by included articles; (2) contacting all corresponding authors of included articles inquiring whether they have any other studies (published or not) that might be eligible for the review; (3) for non-English publications, seeking translations by contacting the primary authors to determine if English versions were available and using translation services (4) searching lists of identified articles (including reviews and editorials) for additional relevant items, and (5) checking reference lists of relevant reviews.

Search results were uploaded to the web-based systematic review software platform Covidence to screen studies. Duplicates were removed and screening was done in two steps. Firstly, titles and abstracts were screened and studies not fulfilling the inclusion criteria were excluded. Where it was uncertain if studies met inclusion criteria, they were retained for the next stage of screening. Secondly, full-text articles were screened out based on inclusion/exclusion criteria. Both stages of screening were completed in parallel by two independent reviewers (ND and TLH) and discrepancies resolved through discussion with a senior reviewer (MOH).

### Eligibility criteria

Population: Participants with diagnosed personality disorders as the primary diagnosis (operationalized by either the DSM or ICD criteria) in a LMIC setting. All settings including outpatient, community and forensic settings were included.

Intervention: Participants receiving any psychosocial or pharmacological intervention

Comparison: Our inclusion criteria encompassed studies that had comparison groups as well as those without. We included studies that measured any

Outcome: Studies that measured any improve their condition, decrease symptom severity, or improve functioning using quantitative measures.

We included the following types of studies:

1. Quantitative studies (case-control, randomized control trial, quasi-randomized trials, case series, single-arm trials, prospective designs) using any validated assessment of illness severity, which is repeated from baseline to endpoint. If multiple outcomes were reported in a study, the primary outcome for inclusion was selected in a hierarchical fashion: the most preferable scale being a clinician-rated assessment tools of severity (example Zanarini Rating Scale for Borderline Personality Disorder; ZAN-BPD), clinical rated tools of general psychopathology (example Brief Psychiatric Rating Scale; BPRS) followed by an assessment of global improvement (example the Clinical Global Impression (CGI); Global Assessment Scale (GAS); or Global Assessment of Function (GAF)) or clinician rated tools of co-morbidity (Hamilton Depression Rating Scale (HAM-D) for depression for example). Where multiple endpoints were reported, this review considered the primary endpoint reported by each study.
2. Studies published in English or, if published in any other language, English translation was sought.
3. Grey literature including dissertations and theses, conference abstracts, and registered completed clinical trials

We excluded:

1) Studies in which PD was not the primary condition
3) Studies conducted in HICs as defined by the World Bank [18]
4) Qualitative studies
5) Articles for which English translations were unavailable

### Critical appraisal

Two researchers (ND and TLH) independently rated the methodological quality of all included studies, and conflicts were resolved by consensus. The Cochrane Risk of Bias tool version 2.0 (RoB 2) was used for the risk of bias assessment of the randomized controlled trials or quasi randomized controlled trials through which studies were categorized on the basis of “low”, “high” and “some concerns”: in the randomization process, deviations from intended interventions, missing outcome data, measurement of the outcome, and selection of the reported result.^16^ An overall quality assessment result was generated based on the RoB 2 algorithm.

For other study designs, such as non-randomized trials, case controls, case series and case studies, we used relevant instruments from the Joanna Briggs Institute (JBI), which provided each study with an overall rating of “include”, “not include” or “seek further information”.^17^

In the original protocol, we intended to use the GRADE approach to assess the overall quality of evidence.^18^ The GRADE approach is designed to grade quality of a body of evidence separately for each patient-important outcome across all the studies in a systematic review. The GRADE approach was not used because the studies that met inclusion criteria did not have consistent enough outcomes or a high enough quality of study design to summarize any results across multiple studies.

### Data extraction

Data extraction was completed by two reviewers (ND and TLH). Conflicts were resolved by discussion between the two reviewers and the senior reviewer (MOH). Data was extracted on the following aspects: study location, participant demographics, sample size, study type, inclusion criteria, exclusion criteria, primary and secondary outcome measures, intervention characteristics, outcome results, drop-out rates, limitations, and adverse effects.

### Synthesis of results

We conducted a narrative synthesis of the studies, grouping them by study type, investigated PD, intervention type, assessment methods, and outcomes. A meta-analysis was not performed due to the heterogeneity of patient populations, interventions, and outcome measurements.

## Results

**Fig 1:**
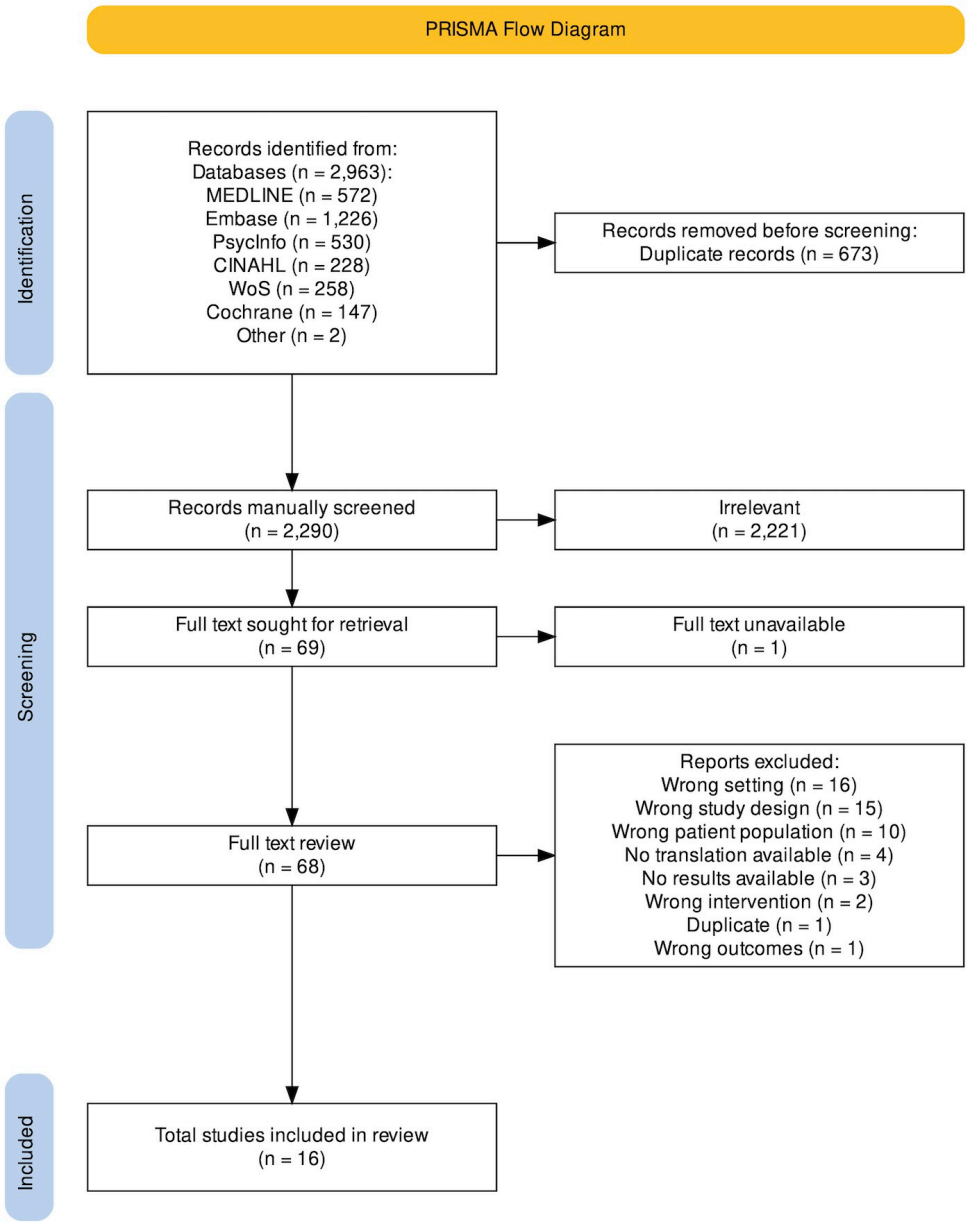
PRISMA flow diagram.

Overall, 2961 studies were identified through a database search and two others were identified by reviewing references. 673 studies were duplicates, and a further 2221 (titles and abstracts) were excluded (Fig 1). Seven studies that made it to full text review were not available in English. For these studies, we contacted the corresponding authors and connected with translation services within our institution. We were able to obtain translations for 3 of these studies. The other four were excluded. After full text review, 16 studies met the inclusion criteria and were included in the review.

Non-randomized experimental studies were the most common study design, making up 8 of the 16 studies. There were only 6 randomized controlled trials (RCTs), which all focused on borderline personality disorder (BPD). There was one cohort study and one semi-experimental study.

### Summary of data extractions

Most of the studies were completed on patients with BPD (n = 15), while only one focused on mixed PDs (n = 1) (Table 1). Selected characteristics of studies included in the systematic review are shown Table 2. Studies were conducted in Iran (n = 8), China (n = 1), Egypt (n = 1), and Mexico (n = 5), or they were done across different LMICs (n = 1). Symptom severity and main outcome measures were mostly measured by self-report questionnaires.

**Table 1:** Studies by type of personality disorder.

PERSONALITY DISORDER	# OF STUDIES
Borderline Personality Disorder (BPD)	15
Mixed Narcissistic Personality Disorder and Antisocial Personality Disorder	1

**Table 2:** Characteristics of included studies.

Study Country	Personality Disorder	Study Design	N =	Intervention	Primary Outcome Measures	Outcomes
**Abdelkarim 2017 Egypt** [35]	BPD	Non-randomized experimental study	40	DBT	DERS, Urine Drug Screen, frequency of alcohol and substance use history	Treatment group showed significantly lower doses of drugs used, Difficulties in Emotion Regulation Scale score. Also, the treatment group showed increased retention in treatment and longer duration of total alcohol abstinence and other drugs of abuse after treatment and four months later.
**Akbari 2009 Iran** [23]	BPD	Randomized controlled trial	40	Lithium and fluoxetine vs. CBT vs Lithium, fluoxetine and CBT	BAI and Impulsivity Checklist	All groups had lower Impulsivity Checklist scores, with the lithium and fluoxetine groups showing the greatest improvement. CBT lowered Beck Anxiety Inventory scales more than pharmacotherapy.
**Reyes-Ortega 2020 Mexico** [30]	BPD	Non-randomized experimental study	65	ACT, DBT, or combined ACT, DBT and FAP	BEST, DERS, AAQ	All three modalities reduced BPD symptom severity and emotional dysregulation, as well as negative interpersonal attachment with no significant difference between control and treatment arms.
**Makvandi 2018 Iran** [31]	BPD	Non-randomized experimental study	36 (12 vs 12 vs 12 controls)	DBT + medication vs. ST + medication vs. medication only	BIS-11	One month after treatment, DBT and Schema Therapy had a significant effect on reducing the Barratt Impulsive Behaviour Questionnaire scores. Also, there was no significant difference between DBT and Schema Therapy.
**Einy 2018 Iran** [32]	BPD	Randomized control trial	45	Mentalization-Based Therapy vs. Cognitive-Analytic therapy sessions.	Bell Object Relations Inventory Questionnaire	Both treatment methods improved the Bell Object Relations Inventory scores.
**Hamid 2020 Iran** [26]	BPD	Non-randomized experimental study	45	DBT vs ST vs control group	BPDSI	DBT and ST were effective in reducing BPDSI scores.
**Mohammadi 2018 Iran** [32]	BPD	Non-randomized experimental study	6	Unified Protocol therapy	Borderline Personality Inventory	The overall remission rate of the participants based on the Borderline Personality Inventory was 0.68 at the end of treatment and 0.34 in the follow-up phase.
**Cuevas 2000 Mexico** [25]	BPD	Cohort study	19	Psychodynamic psychotherapy	Clarkin’s Borderline Personality Disorder and Global Activity Assessment Scale.	Almost 60% of the sample no longer met criteria for BPD after 9 months of therapy based on Clarkin’s Borderline Personality Disorder and Global Activity Assessment Scale scores
**Wang 2008 China** [22]	NPD and ASPD	Non-randomized experimental study	110 total (30 healthy volunteers, 50 people with ASPD, 30 with NPD)	Family Behaviour Therapy	Plutchik-Van Praag Depression Inventory	Most self-reported symptoms and Plutchik-Van Praag Depression Inventory scores were significantly lowered in both patient groups.
**Kamalabadi 2012 Iran** [29]	BPD	Randomized control trial	30	14 weekly sessions of couples DBT	BPDSI, PRQC, GHQ-28	The treatment group had lower scores on the BPDSI and 3 subscales of the GHQ-28, and higher scores on 5 subscales of the PRQC one month after the end of treatment.
**Majdara 2021 Iran** [34]	BPD	Randomized control trial	30	Pharmacotherapy and DDP	BEST	Reduction of BEST scores at post-test and follow up. 67% achieved at least 25% improvement in BEST scores after 12 months
**Reyes-Lopez 2018 Mexico** [20]	BPD	Non-randomized experimental study	29	15 sessions of rTMS over the right (1 Hz, n = 15) or left (5 Hz, n = 14) dorsolateral prefrontal cortex	CGI-BPD, BEST, BDI, HAM-A, and Barratt Impulsiveness Scale	Intragroup comparison showed significant (p < 0.05) reductions in every psychopathologic domain of the CGI-BPD and in the total scores of all scales in both groups.
Zanarini 2011 Multiple countries* [24]	BPD	Randomized control trial	451 (olanzapine 2.5 n = 150, olanzapine 5–10 mg n 148, placebo n = 153)	Olanzapine 2.5 mg vs olanzapine 5–10 mg vs placebo	ZAN-BPD	Olanzapine 5–10 mg/d significantly decreased Zanarini Rating Scale for BPD total score relative to placebo respectively. Rate of response was significantly higher for olanzapine 5–10 mg/d versus olanzapine 2.5 mg/d vs placebo.
**Calderón-Moctezuma 2021, Mexico** [28]	BPD	Randomized control trial	18 (9 intervention, 9 control)	rTMS 5-Hz rTMSm five days a week for three weeks	BSL, CGI-BPD, and BEST	Statistical reduction in CGI-BPD and BEST scores
**Keshvari 2021, Iran** [21]	BPD	Semi-experimental	32 (16 intervention, 16 control)	Mindfulness-based psychotherapy x 8 weekly sessions (treatment group) vs DBT (control group) vs no intervention	BAI and Jones Irrational Beliefs Questionnaire	Statistically significantly decrease in both anxiety and irrational beliefs in posttest and follow up.
**Orduna 2022, Mexico** [19]	BPD	Case series	7	Culturally adapted, Spanish-translated manual assisted cognitive therapy (5–6 weekly sessions)	Weekly self-report of NSSI behavior	Most participants demonstrated a significant reduction in NSSI

Acceptance and Commitment Therapy (ACT), Adult Attachment Questionnaire (AAQ), Antisocial personality disorder (ASPD), Barratt Impulsive Behaviour Questionnaire (BIS-11), Beck Anxiety Inventory (BAI), Beck Depression Inventory (BDI), Borderline Personality Disorder (BPD), Borderline Personality Disorder Severity Index (BPDSI), Borderline Symptoms List (BSL), Borderline Evaluation of Severity over Time (BEST), Clinical Global Impression for BPD (CGI-BPD), Cognitive Behavioural Therapy (CBT), Dialectical Behavioural Therapy (DBT), Diagnostic and Statistical Manual of Mental Disorders—IV (DSM-IV), Difficulties in Emotion Regulation Scale (DERS), Dynamic Deconstrucive Psychotherapy (DDP), Functional Analytic Psychotherapy (FAP), General Mental Health Questionnaire (GHQ-28), Hamilton Anxiety Rating Scale (HAM-A), Narcissistic Personality Disorder (NPD), Non-suicidal self-injury (NSSI), Perceived Relationship Quality Components Inventory (PRQC), Repetitive Transmagnetic Stimulation (rTMS), Schema Therapy (ST), Zanarini Rating Scale for BPD (ZAN-BPD).

* LMICs from this study include Turkey, Peru, Argentina. Other countries included in study were the United States, Italy, Poland, Romania, and Chile, which are high income countries as per the World Bank. Venezuela is not classified as per the World Bank.

### Interventions for BPD

Most of the interventions for BPD were psychotherapy based (n = 10), while one focused on pharmacological interventions only and three studies combined pharmacological interventions and psychotherapy. There were two brain therapeutic trials which studied rTMS.

The type of psychotherapy and frequency differed greatly between studies from 12 weeks to 2 years. Positive outcomes were noted for dialectical behavioural therapy (DBT), couples DBT, schema therapy (ST), unified protocol therapy, cognitive behavioural therapy (CBT), acceptance and commitment therapy (ACT), functional analytic therapy (FAP), mentalization-based therapy, cognitive-analytic therapy, psychodynamic psychotherapy, deconstructive dynamic therapy (DDP), and mindfulness-based therapy. There was only one study that noted using a cultural adapted psychotherapy modality [19]. Positive results were noted for treating BPD with lithium, fluoxetine, olanzapine, and with repetitive Transcranial Magnetic Stimulation (rTMS).

As demonstrated in Table 2, most studies focused on self-report measures and questionnaires. Most of these questionnaires were specific to BPD and core BPD symptoms while four studies included depression and anxiety symptoms as primary outcome measures [20–23].

### Interventions for other PDs

All studies focused on BPD except for one non-randomized experimental study that included individuals with narcissistic personality disorder (NPD) and antisocial personality disorder (ASPD) [22]. In this study, the treatment was 8 sessions of family behavior therapy, after which symptoms were assessed using self-report scales. None of the studies involved treatments for personality disorders other than BPD, NPD and ASPD.

### Adverse effects, acceptability, suicidal ideation

The dropout rates for the mixed PD study were 56% for ASPD and 53.3% for NPD population [22]. Most studies did not comment on adherence in their articles. In the olanzapine trial, they reported over a 60% adherence rate in all groups and the intervention arms were similar to the placebo groups [24]. For long-term psychological interventions like psychodynamic psychotherapy, only 21% of the initial participants attended sessions for the entire two years [25].

For limitations, many identified small samples sizes [19,20,23,26–28] and durations of follow-up [22,23,26,29], lack of randomization, lack of a control group, or study methodology was identified as a limitation in three of the studies [20,22,30]. Three studies mentioned their lack of long-term follow up as a limitation [31–33]. In the Hamid *et al*. paper that focused on DBT and schema therapy, they had no female participants [26]. Multiple studies reported limited generalizability of the sample population, with Orduna *et al*. explaining that their sample was a highly motivated group, which is less generalizable to the greater personality disorder population [19,33]. Although self-report scales were the primary outcome measures in most studies, only one study acknowledged this as a limitation [34].

Abdelkarim *et al*., which studied DBT for BPD and comorbid substance use reported reduced suicide attempts in their treatment arm. [35] The olanzapine trial for BPD reported that two patients in the placebo group attempted suicide [36]. Orduna *et al*. was the only study that focused on non-suicidal self injury (NSSI) as a primary outcome measure [19]. The other studies did not report suicidal ideation, suicide behaviors, or NSSI as outcome measures.

For adverse effects, Cuevas *et al*., noted that they had one participant who was participating in psychodynamic psychotherapy who relapsed on substances and was subsequently hospitalized [25]. In the RCT studying olanzapine in BPD, about 5% of patients reported emergent adverse events in the treatment group. Common side effects like somnolence, increased appetite and weight gain were also reported at an increased rate in the treatment groups [36].

### Critical appraisal of included studies

In terms of the hierarchy of evidence, our sample included six randomized controlled trials, one cohort study, one case series, and eight non-randomized experimental studies. By virtue of not being randomized, the ten studies that were not RCTs have a higher risk of selection bias. For the non-randomized studies, five studies reported vague primary outcomes. Almost all studies clearly defined their intervention and post intervention clinical condition. Overall, using the Joanna Briggs Institute tools, all studies were deemed suitable to “include” in our analysis [37]. Of the RCTs, three were determined to have a high risk of bias, one study had some concerns, and two were found to have a low risk of bias using the Cochrane Risk of Bias Tool 2.0 (Table 3) [38].

**Table 3:** Cochrane Risk of Bias for included randomized control trials.

	Randomization process	Deviations from the intended interventions	Missing outcome data	Measurement of the outcome	Selection of the reported result	Overall
**Akbari, 2009** [23]	+	+	+	!	+	Some concerns
**Einy, 2018** [32]	+	+	+	+	-	High risk
**Majdara. 2021** [34]	+	+	+	+	+	Low risk
**Zanarini, 2011** [36]	+	+	+	+	+	Low risk
**Kamalabadi, 2012** [29]	+	+	+	+	-	High risk
**Calderón-Moctezuma, 2021** [28]	+	+	+	!	-	High Risk

Low risk (+), some concerns (!), high risk (-).

Of the sixteen studies included, twelve do not mention any source of funding and one described receiving no funding. Only three studies specifically mention being funded; one by several government departments in China [22], one by a government agency in Mexico and an affiliated university [28], and the olanzapine trial was funded by Eli Lilly and Company [36]. The only study that reported possible conflicts of interest for the authors was the olanzapine trial funded by Eli Lilly and Company [36]. Common limitations highlighted by our critical appraisal were low sample size, limited follow-up, high dropout, and lack of randomization or absence of control group.

### Meta-analysis

Due to a large heterogeneity in participant, interventions, comparators, and outcomes (PICO) as well as concerned raised regarding risk of bias in multiple included studies, the selected studies were not pooled for meta-analysis [39,40]. Carrying out a meta-analysis poses a high risk of skewing data results, rather than increasing their statistical power. The six RCTs for BPD all had different interventions and outcome measures.

## Discussion

There is a paucity of data on interventions for PDs in LMICs despite the large burden of disease in these populations. Our review only identified 16 studies investigating interventions for PDs in LMICs. In comparison, systematic reviews focused on methodologically rigorous clinical trials in PDs in HICs have identified over 20 RCTs investigating specialized psychotherapies for BPD alone.^31^ It is important to highlight that there were no studies conducted in South Asian or African countries, which significantly limits our understanding of effective interventions in these populous regions. Our review highlights the need for more high quality and methodologically rigorous clinical trials of interventions for PDs in LMICs.

The studies reviewed largely favor psychotherapy treatments for personality disorders, which is in keeping with research conducted in HICs [41]. Only one study addressed cultural adaptation of psychotherapy, which is a crucial step in meeting the needs of diverse populations [19,42]. Research on pharmacological interventions and neurostimulation (rTMS) is limited. Despite there being more research on BPD, it was not sufficient to make strong clinical recommendations for treatment of BPD in LMICs. With even scarcer literature for other PDs, no clinical recommendations could be firmly made from the results of our review.

This systematic review, while comprehensive, carries certain limitations that warrant careful consideration. The considerable heterogeneity among the studies regarding interventions, study designs, and outcome measures forestalled the possibility of conducting a meta-analysis. This heterogeneity arises from diverse elements such as the variety of personality disorders examined, the differing intervention techniques, the unique cultural contexts of the LMICs studied, and a broad range of outcome measures utilized, some of which were specifically designed for individual studies.

These factors not only contribute to the inability to perform a meta-analysis but also complicate direct comparison of results among the included studies, or to equivalent studies performed in high-income countries. Frequently, studies inferred improvement through indirect indicators such as reduction in comorbid behaviors, like cessation of drug use or decrease in anxiety and depression scores, rather than direct change in core PD symptom domains or functioning. This can be viewed as another layer of heterogeneity that hampers our ability to draw consistent conclusions.

In addition, many of the studies documented substantial improvement in symptoms over a relatively brief follow-up period, raising questions about the long-term efficacy and stability of the reported gains.

Lastly, we acknowledge the language limitation in our review. Four studies were excluded from our review because English versions were not available, which may have led to English-language bias [43].

This review emphasizes the need for more investment from funding bodies towards methodologically rigorous clinical trials examining the clinical efficiency of interventions for PDs in LMICs. Adequate investment for research in LMICs would facilitate the generation of reliable data needed to inform evidence -based treatment guidelines and the development of patient-centered mental health services to meet the needs of individuals within these settings. We recommend that future trials used standardized outcome measures to provide more opportunities for meta-analysis. Most of the PD research in LMICs has focused on BPD; we emphasize that research on other PDs, like ASPD, should not be ignored as these disorders are also associated with distress, disability and significant societal burden [9]. Future research should aim to culturally-adapt psychotherapy interventions that have evidence for clinical efficacy for treating PDs in HICs to the local contexts in which they will be delivered. Adaptation should be followed by well-designed clinical trials to establish clinical efficacy within these settings. Given the relatively low resources available for mental health service delivery in most LMICs, shifting the focus to more innovative approaches to intervention delivery with scalability in mind should be prioritized. [6] Developing digital psychosocial interventions that are culturally adapted to the local context could help bridge the gap in mental health treatment in LMICs. In HICs, digital psychotherapy interventions have been demonstrated to be effective for several major mental health disorders and may be more accessible and cost-effective than traditional costly psychotherapy [44].

## Conclusion

Less than 10% of mental health research is conducted in LMICs despite most individuals suffering from mental health disorders reside in these settings [13]. The Lancet commission on global mental health recognizes that that supporting global mental health research is a crucial part of enhancing the delivery of evidence-based care in LMICs [6]. Prioritizing funding for this research is also essential [6]. While the call for evidence-based interventions for mental disorders in LMICs has been growing, PDs remain underrecognized, service for affected individuals is underfunded, and subsequently undertreated despite their high morbidity and mortality [9]. We recommend further research on PDs in LMICs given the high prevalence, high morbidity and lack of evidence-based interventions in these settings.

## Supporting information

S1 File PRISMA checklist. (PDF)

S2 File Search strategy. (PDF)

S3 File Cochrane risk of bias for included randomized control trials data. (XLSM)

S4 File Joanna Briggs risk of bias data. (CSV)

S5 File Data extraction data. (CSV)
