# Supplementary material for: Psychosocial and pharmacological interventions for personality disorders in low- and middle-income countries: A systematic review
Source: PLOS Glob Public Health. 2023 Nov 3;3(11):e0002485. doi: 10.1371/journal.pgph.0002485 (PMC10624326; doi:10.1371/journal.pgph.0002485)
Supplement: S2 File — (PDF) [file pgph.0002485.s002.pdf]

Supplementary Material 2

Ovid MEDLINE: Epub Ahead of Print, In-Process & Other Non-Indexed Citations, Ovid MEDLINE® Daily and Ovid MEDLINE® <1946-Present>

1 exp Personality Disorders/
2 personality disorder*.ti,ab,kf.
3 ((paranoid or schizoid or schizotypal or antisocial or anti-social or asocial or dissocial or borderline or histrionic or narcissistic or avoidant or dependent or obsessive compulsive) adj2 personalit*).ti,ab,kf.
4 ((passive-aggressive or passive-depressive or hysterical or compulsive or inadequate or explosive or asthenic or sadis* or masochis* or self-defeating or negativistic or depressive) adj2 personalit*).ti,ab,kf.
5 cluster A.ti,ab,kf.
6 cluster B.ti,ab,kf.
7 cluster C.ti,ab,kf.
8 (BPD not (biparietal diameter or bronchopulmonary dysplasia)).ti,ab,kf.
9 sociopath*.ti,ab,kf.
10 (psychopath or psychopaths or psychopathy or psychopathic).ti,ab,kf.
11 hysteria.ti,ab,kf.
12 Developing Countries/
13 ((low* income or middle income or developing or emerging) adj3 (country or countries or nation or nations or econom*)).ti,ab,kf.
14 developing world.ti,ab,kf.
15 (third world or second world).ti,ab,kf.
16 global south.ti,ab,kf.
17 LMIC*.ti,ab,kf.
18 ((Middle East* or mideast* or mid-east* or Africa or Central America* or South America* or South* Asia* or Southeast* Asia* or South east* asia* or Afghanistan or Albania* or Algeria* or American Samoa* or Angola* or Argentin* or Armenia* or Azerbaijan* or Azeri or Bangladesh* or Belarus* or Belize* or Benin* or Bhutan* or Bolivia* or Bosnia* or Herzegovin* or Botswana* or Motswana* or Brazil* or Bulgaria* or Burkina Faso or Burkinabe or Burundi* or Cape Verde* or Cabo Verde* or Cambodia* or Cameroon* or Central African Republic or Central African or Chad or Chadian or China or Chinese or Colombia* or Comoros or Comorian or Congo* or Costa Rica* or "Cote D'ivoire" or Ivorian or Cuba* or Djibouti* or Dominica* or Dominican Republic or Ecuador* or Egypt* or El Salvador* or Equatorial Guinea or Equatoguinean or Eritrea* or Eswatini or Emaswati or Ethiopia* or Fiji* or Gabon* or Gabonaise or Gambia* or Georgia* or Ghana* or Grenad* or Guatemala* or Guinea or Guinea Bissau or Bissau-Guinean or Guyan* or Haiti* or Hondura* or India* or Indonesia* or Iran* or Iraq* or Jamaica* or Jordan* or Kazakhstan* or Kenya* or

Kiribati or Korea* or Kosov* or Kyrgyz* or Lao or Laos or Laotian or Lebanon or Lebanese or Lesotho or Mosotho or Basotho or Liberia* or Libya* or Madagascar or Malagasy or Malawi* or Malaysia* or Maldiv* or Mali or Malian or Marshall Islands or Marshallese or Mauritania* or Mexic* or Micronesia* or Moldova* or Mongolia* or Montenegr* or Morocc* or Mozambi* or Myanma* or Burmese or Namibia* or Nepal* or Nicaragua* or Niger* or Nigeria* or Macedonia* or Pakistan* or Papua New Guinea* or Paraguay* or Peru* or Philippines or Filipin* or Russian Federation or Russia* or Rwand* or Samoa* or Sao Tome* or Santomean or Principe or Senegal* or Serbia* or Sierra Leone* or Solomon Island* or Somali* or South Africa* or South Sudan* or Sri Lanka* or St* Lucia* or Saint Lucia* or St* Vincent or Saint Vincent or Vincentian* or Swaziland or Grenadin* or Sudan* or Surinam* or Syria* or Tajikistan* or Tanzania* or Thai* or Timor* or Togo or Togolese or Tonga* or Tunisia* or Turk* or Turkmenistan or Tuvalu* or Uganda* or Ukrain* or Uzbek* or Vanuatu* or Venezuela* or Vietnam* or West Bank or Gaza* or palestin* or "occupied territor*" or Yemen* or Zambia* or Zimbabwe*) not american indian*).ti,ab,kf,hw,jn.

19 (Randomized Controlled Trial or Controlled Clinical Trial or Pragmatic Clinical Trial or Equivalence Trial or Clinical Trial, Phase III).pt.

20 Randomized Controlled Trial/

21 exp Randomized Controlled Trials as Topic/

22 Controlled Clinical Trial/

23 exp Controlled Clinical Trials as Topic/

24 Random Allocation/

25 Double-Blind Method/

26 Single-Blind Method/

27 Placebos/

28 Control Groups/

29 (random* or sham or placebo*).ti,ab,hw,kf.

30 ((singl* or doubl*) adj (blind* or dumm* or mask*)).ti,ab,hw,kf.

31 ((tripl* or trebl*) adj (blind* or dumm* or mask*)).ti,ab,hw,kf.

32 (control* adj3 (study or studies or trial* or group*)).ti,ab,kf.

33 (Nonrandom* or non random* or non-random* or quasi-random* or quasirandom*).ti,ab,hw,kf.

34 allocated.ti,ab,hw.

35 ((open label or open-label) adj5 (study or studies or trial*)).ti,ab,hw,kf.

36 ((equivalence or superiority or non-inferiority or noninferiority) adj3 (study or studies or trial*)).ti,ab,hw,kf.

37 (pragmatic study or pragmatic studies).ti,ab,hw,kf.

38 ((pragmatic or practical) adj3 trial*).ti,ab,hw,kf.

39 ((quasiexperimental or quasi-experimental) adj3 (study or studies or trial*)).ti,ab,hw,kf.

40 (phase adj3 (III or "3") adj3 (study or studies or trial*)).ti,hw,kf.

41 exp Drug Therapy/

42	case reports/
43	editorial/
44	comment/
45	letter/
46	systematic review/
47	Systematic Reviews as Topic/
48	meta-analysis/
49	meta-analysis as topic/
50	("systematic review" or "scoping review" or "review of reviews" or meta-analy* or metaanaly*).ti,ab,kf.
51	or/1-11
52	or/12-18
53	or/19-50
54	51 and 52 and 53

Embase Classic+Embase <1947 to 2021 January 05>	
1	exp personality disorder/
2	personality disorder*.ti,ab,kw.
3	((paranoid or schizoid or schizotypal or antisocial or anti-social or asocial or dissocial or borderline or histrionic or narcissistic or avoidant or dependent or obsessive compulsive) adj2 personalit*).ti,ab,kw.
4	((passive-aggressive or passive-depressive or hysterical or compulsive or inadequate or explosive or asthenic or sadis* or masochis* or self-defeating or negativistic or depressive) adj2 personalit*).ti,ab,kw.
5	cluster A.ti,ab,kw.
6	cluster B.ti,ab,kw.
7	cluster C.ti,ab,kw.
8	(BPD not (biparietal diameter or bronchopulmonary dysplasia)).ti,ab,kw.
9	sociopath*.ti,ab,kw.
10	(psychopath or psychopaths or psychopathy or psychopathic).ti,ab,kw.
11	(hysteria or hysterical).ti,ab,kw.
12	developing country/
13	((low* income or middle income or developing or emerging) adj3 (country or countries or nation or nations or econom*)).ti,ab,kw.
14	developing world.ti,ab,kw.
15	(third world or second world).ti,ab,kw.
16	global south.ti,ab,kw.
17	LMIC*.ti,ab,kw.
18	((Middle East* or mideast* or mid-east* or Africa or Central America* or South America* or South* Asia* or Southeast* Asia* or South east* asia* or Afghanistan or Albania* or Algeria* or American Samoa* or Angola* or Argentin* or Armenia* or Azerbaijan* or Azeri or Bangladesh* or Belarus* or Belize* or Benin* or Bhutan* or Bolivia* or Bosnia* or Herzegovin* or Botswana* or Motswana* or Brazil* or Bulgaria* or Burkina Faso or Burkinabe or Burundi* or Cape Verde* or Cabo Verde* or Cambodia* or Cameroon* or Central African Republic or Central African or Chad or Chadian or China or Chinese or Colombia* or Comoros or Comorian or Congo* or Costa Rica* or "Cote D'ivoire" or Ivorian or Cuba* or Djibouti* or Dominica* or Dominican Republic or Ecuador* or Egypt* or El Salvador* or Equatorial Guinea or Equatoguinean or Eritrea* or Eswatini or Emaswati or Ethiopia* or Fiji* or Gabon* or Gabonaise or Gambia* or Georgia* or Ghana* or Grenad* or Guatemala* or Guinea or Guinea Bissau or Bissau-Guinean or Guyan* or Haiti* or Hondura* or India* or Indonesia* or Iran* or Iraq* or Jamaica* or Jordan* or Kazakhstan* or Kenya* or Kiribati or Korea* or Kosov* or Kyrgyz* or Lao or Laos or Laotian or Lebanon or Lebanese or Lesotho or Mosotho or Basotho or Liberia* or Libya* or Madagascar or

Malagasy or Malawi* or Malaysia* or Maldiv* or Mali or Malian or Marshall Islands or Marshallese or Mauritania* or Mexic* or Micronesia* or Moldova* or Mongolia* or Montenegr* or Morocc* or Mozambi* or Myanma* or Burmese or Namibia* or Nepal* or Nicaragua* or Niger* or Nigeria* or Macedonia* or Pakistan* or Papua New Guinea* or Paraguay* or Peru* or Philippines or Filipin* or Russian Federation or Russia* or Rwand* or Samoa* or Sao Tome* or Santomean or Principe or Senegal* or Serbia* or Sierra Leone* or Solomon Island* or Somali* or South Africa* or South Sudan* or Sri Lanka* or St* Lucia* or Saint Lucia* or St* Vincent or Saint Vincent or Vincentian* or Swaziland or Grenadin* or Sudan* or Surinam* or Syria* or Tajikistan* or Tanzania* or Thai* or Timor* or Togo or Togolese or Tonga* or Tunisia* or Turk* or Turkmenistan or Tuvalu* or Uganda* or Ukrain* or Uzbek* or Vanuatu* or Venezuela* or Vietnam* or West Bank or Gaza* or palestin* or "occupied territor*" or Yemen* or Zambia* or Zimbabwe*) not american indian*).ti,ab,kw,hw,jn.
19 randomized controlled trial/
20 randomized controlled trial (topic)/
21 controlled clinical trial/
22 controlled clinical trial (topic)/
23 randomization/
24 double blind procedure/
25 single blind procedure/
26 placebo/
27 control group/
28 (random* or sham or placebo*).ti,ab,hw,kw.
29 ((singl* or doubl*) adj (blind* or dumm* or mask*)).ti,ab,hw,kw.
30 ((tripl* or trebl*) adj (blind* or dumm* or mask*)).ti,ab,hw,kw.
31 (control* adj3 (study or studies or trial* or group*)).ti,ab,kw.
32 (Nonrandom* or non random* or non-random* or quasi-random* or quasirandom*).ti,ab,hw,kw.
33 allocated.ti,ab,hw.
34 ((open label or open-label) adj5 (study or studies or trial*)).ti,ab,hw,kw.
35 ((equivalence or superiority or non-inferiority or noninferiority) adj3 (study or studies or trial*)).ti,ab,hw,kw.
36 (pragmatic study or pragmatic studies).ti,ab,hw,kw.
37 ((pragmatic or practical) adj3 trial*).ti,ab,hw,kw.
38 ((quasiexperimental or quasi-experimental) adj3 (study or studies or trial*)).ti,ab,hw,kw.
39 (phase adj3 (III or "3") adj3 (study or studies or trial*)).ti,hw,kw.
40 exp drug therapy/
41 case report/
42 editorial/
43 letter/
44 systematic review/

45	("systematic review" or "scoping review" or "review of reviews").ti,ab,kw.
46	or/1-11
47	or/12-18
48	or/19-45
49	46 and 47 and 48
50	46 and 47
51	limit 50 to (editorial or letter)
52	limit 50 to (randomized controlled trial or controlled clinical trial)
53	49 or 51 or 52
54	limit 53 to medline
55	53 not 54

APA PsycInfo <1806 to December Week 4 2020>

1 exp personality disorders/
2 personality disorder*.ti,ab,id.
3 ((paranoid or schizoid or schizotypal or antisocial or anti-social or asocial or dissocial or borderline or histrionic or narcissistic or avoidant or dependent or obsessive compulsive) adj2 personalit*).ti,ab,id.
4 ((passive-aggressive or passive-depressive or hysterical or compulsive or inadequate or explosive or asthenic or sadis* or masochis* or self-defeating or negativistic or depressive) adj2 personalit*).ti,ab,id.
5 cluster A.ti,ab,id.
6 cluster B.ti,ab,id.
7 cluster C.ti,ab,id.
8 (BPD not (biparietal diameter or bronchopulmonary dysplasia)).ti,ab,id.
9 sociopath*.ti,ab,id.
10 (psychopath or psychopaths or psychopathy or psychopathic).ti,ab,id.
11 (hysteria or hysterical).ti,ab,id.
12 developing countries/
13 emerging economies/
14 ((low* income or middle income or developing or emerging) adj3 (country or countries or nation or nations or econom*)).ti,ab,id.
15 developing world.ti,ab,id.
16 (third world or second world).ti,ab,id.
17 global south.ti,ab,id.
18 LMIC*.ti,ab,id.
19 ((Middle East* or mideast* or mid-east* or Africa or Central America* or South America* or South* Asia* or Southeast* Asia* or South east* asia* or Afghanistan or Albania* or Algeria* or American Samoa* or Angola* or Argentin* or Armenia* or Azerbaijan* or Azeri or Bangladesh* or Belarus* or Belize* or Benin* or Bhutan* or Bolivia* or Bosnia* or Herzegovin* or Botswana* or Motswana* or Brazil* or Bulgaria* or Burkina Faso or Burkinabe or Burundi* or Cape Verde* or Cabo Verde* or Cambodia* or Cameroon* or Central African Republic or Central African or Chad or Chadian or China or Chinese or Colombia* or Comoros or Comorian or Congo* or Costa Rica* or "Cote D'ivoire" or Ivorian or Cuba* or Djibouti* or Dominica* or Dominican Republic or Ecuador* or Egypt* or El Salvador* or Equatorial Guinea or Equatoguinean or Eritrea* or Eswatini or Emaswati or Ethiopia* or Fiji* or Gabon* or Gabonaise or Gambia* or Georgia* or Ghana* or Grenad* or Guatemala* or Guinea or Guinea Bissau or Bissau-Guinean or Guyan* or Haiti* or Hondura* or India* or Indonesia* or Iran* or Iraq* or Jamaica* or Jordan* or Kazakhstan* or Kenya* or Kiribati or Korea* or Kosov* or Kyrgyz* or Lao or Laos or Laotian or Lebanon or

Lebanese or Lesotho or Mosotho or Basotho or Liberia* or Libya* or Madagascar or Malagasy or Malawi* or Malaysia* or Maldiv* or Mali or Malian or Marshall Islands or Marshallese or Mauritania* or Mexic* or Micronesia* or Moldova* or Mongolia* or Montenegr* or Morocc* or Mozambi* or Myanma* or Burmese or Namibia* or Nepal* or Nicaragua* or Niger* or Nigeria* or Macedonia* or Pakistan* or Papua New Guinea* or Paraguay* or Peru* or Philippines or Filipin* or Russian Federation or Russia* or Rwand* or Samoa* or Sao Tome* or Santomean or Principe or Senegal* or Serbia* or Sierra Leone* or Solomon Island* or Somali* or South Africa* or South Sudan* or Sri Lanka* or St* Lucia* or Saint Lucia* or St* Vincent or Saint Vincent or Vincentian* or Swaziland or Grenadin* or Sudan* or Surinam* or Syria* or Tajikistan* or Tanzania* or Thai* or Timor* or Togo or Togolese or Tonga* or Tunisia* or Turk* or Turkmenistan or Tuvalu* or Uganda* or Ukrain* or Uzbek* or Vanuatu* or Venezuela* or Vietnam* or West Bank or Gaza* or palestin* or "occupied territor*" or Yemen* or Zambia* or Zimbabwe*) not american indian*).ti,ab,id,hw,jn.

20 exp randomized controlled trials/
21 placebo/
22 experiment controls/
23 (random* or sham or placebo*).ti,ab,hw,id.
24 ((singl* or doubl*) adj (blind* or dumm* or mask*)).ti,ab,hw,id.
25 ((tripl* or trebl*) adj (blind* or dumm* or mask*)).ti,ab,hw,id.
26 (control* adj3 (study or studies or trial* or group*)).ti,ab,id.
27 (Nonrandom* or non random* or non-random* or quasi-random* or quasirandom*).ti,ab,hw,id.
28 allocated.ti,ab,hw,id.
29 ((open label or open-label) adj5 (study or studies or trial*)).ti,ab,hw,id.
30 ((equivalence or superiority or non-inferiority or noninferiority) adj3 (study or studies or trial*)).ti,ab,hw,id.
31 (pragmatic study or pragmatic studies).ti,ab,hw,id.
32 ((pragmatic or practical) adj3 trial*).ti,ab,hw,id.
33 ((quasiexperimental or quasi-experimental) adj3 (study or studies or trial*)).ti,ab,hw,id.
34 (phase adj3 (III or "3") adj3 (study or studies or trial*)).ti,hw,id.
35 drug therapy/
36 case report/
37 systematic review/
38 ("systematic review" or "scoping review" or "review of reviews" or meta-analy* or metaanaly*).ti,ab,id.
39 or/1-11
40 or/12-19
41 or/20-38
42 39 and 40
43 limit 42 to ("0300 clinical trial" or "0430 followup study" or "0450 longitudinal study" or "0453 retrospective study" or 2100 treatment outcome)

44	limit 42 to "0300 clinical trial"
45	limit 42 to "0200 clinical case study"
46	limit 42 to ("column/opinion" or "comment/reply" or editorial or letter)
47	limit 42 to ("0830 systematic review" or 1200 meta analysis)
48	43 or 44 or 45 or 46 or 47
49	39 and 40 and 41
50	48 or 49

CINAHL – Wednesday, January 06, 2021	
S1	(MH "Personality Disorders+")
S2	personality disorder*
S3	((paranoid or schizoid or schizotypal or antisocial or anti-social or asocial or dissocial or borderline or histrionic or narcissistic or avoidant or dependent or obsessive compulsive) N2 personalit*)
S4	((passive-aggressive or passive-depressive or hysterical or compulsive or inadequate or explosive or asthenic or sadis* or masochis* or self-defeating or negativistic or depressive) N2 personalit*)
S5	("cluster A" and personality)
S6	"cluster B"
S7	"cluster C"
S8	(BPD not (biparietal diameter or bronchopulmonary dysplasia))
S9	sociopath*
S10	(psychopath or psychopaths or psychopathy or psychopathic)
S11	(hysteria or hysterical)
S12	(MH "Developing Countries")
S13	((low* income or middle income or developing or emerging) N3 (country or countries or nation or nations or econom*))
S14	developing world
S15	(third world or second world)
S16	global south
S17	LMIC*
S18	((Middle East* or mideast* or mid-east* or Africa or Central America* or South America* or South* Asia* or Southeast* Asia* or South east* asia* or Afghanistan or Albania* or Algeria* or American Samoa* or Angola* or Argentin* or Armenia* or Azerbaijan* or Azeri or Bangladesh* or Belarus* or Belize* or Benin* or Bhutan* or Bolivia* or Bosnia* or Herzegovin* or Botswana* or Motswana* or Brazil* or Bulgaria* or Burkina Faso or Burkinabe or Burundi* or Cape Verde* or Cabo Verde* or Cambodia* or Cameroon* or Central African Republic or Central African or Chad

	or Chadian or China or Chinese or Colombia* or Comoros or Comorian or Congo* or Costa Rica* or "Cote D'ivoire" or Ivorian or Cuba* or Djibouti* or Dominica* or Dominican Republic or Ecuador* or Egypt* or El Salvador* or Equatorial Guinea or Equatoguinean or Eritrea* or Eswatini or Emaswati or Ethiopia* or Fiji* or Gabon* or Gabonaise or Gambia* or Georgia* or Ghana* or Grenad* or Guatemala* or Guinea or Guinea Bissau or Bissau-Guinean or Guyan* or Haiti* or Hondura* or India* or Indonesia* or Iran* or Iraq* or Jamaica* or Jordan* or Kazakhstan* or Kenya* or Kiribati or Korea* or Kosov* or Kyrgyz* or Lao or Laos or Laotian or Lebanon or Lebanese or Lesotho or Mosotho or Basotho or Liberia* or Libya* or Madagascar or Malagasy or Malawi* or Malaysia* or Maldiv* or Mali or Malian or Marshall Islands or Marshallese or Mauritania* or Mexic* or Micronesia* or Moldova* or Mongolia* or Montenegr* or Morocc* or Mozambi* or Myanma* or Burmese or Namibia* or Nepal* or Nicaragua* or Niger* or Nigeria* or Macedonia* or Pakistan* or Papua New Guinea* or Paraguay* or Peru* or Philippines or Filipin* or Russian Federation or Russia* or Rwand* or Samoa* or Sao Tome* or Santomean or Principe or Senegal* or Serbia* or Sierra Leone* or Solomon Island* or Somali* or South Africa* or South Sudan* or Sri Lanka* or St* Lucia* or Saint Lucia* or St* Vincent or Saint Vincent or Vincentian* or Swaziland or Grenadin* or Sudan* or Surinam* or Syria* or Tajikistan* or Tanzania* or Thai* or Timor* or Togo or Togolese or Tonga* or Tunisia* or Turk* or Turkmenistan or Tuvalu* or Uganda* or Ukrain* or Uzbek* or Vanuatu* or Venezuela* or Vietnam* or West Bank or Gaza* or palestin* or "occupied territor*" or Yemen* or Zambia* or Zimbabwe*) not american indian*)
S19	Randomized Controlled Trial or Controlled Clinical Trial or Pragmatic Clinical Trial or Equivalence Trial
S20	(MH "Randomized Controlled Trials+")
S21	(MH "Random Assignment")
S22	(MH "Double-Blind Studies")
S23	(MH "Single-Blind Studies")
S24	(MH "Placebos")
S25	(MH "Control Group")
S26	(random* or sham or placebo*)
S27	((singl* or doubl*) N1 (blind* or dumm* or mask*))
S28	((tripl* or trebl*) N1 (blind* or dumm* or mask*))
S29	(control* N3 (study or studies or trial* or group*))
S30	allocated
S31	((open label or open-label) N5 (study or studies or trial*))
S32	((equivalence or superiority or non-inferiority or noninferiority) N3 (study or studies or trial*))
S33	(MH "Drug Therapy+")
S34	(MH "Case Studies")
S35	editorial or opinion or commentary or "letter to the editor"
S36	S1 OR S2 OR S3 OR S4 OR S5 OR S6 OR S7 OR S8 OR S9 OR S10 OR S11

S37	(S12 OR S13 OR S14 OR S15 OR S16 OR S17 OR S18)
S38	(S19 OR S20 OR S21 OR S22 OR S23 OR S24 OR S25 OR S26 OR S27 OR S28 OR S29 OR S30 OR S31 OR S32 OR S33 OR S34 OR S35)
S39	systematic review or meta-analysis or metaanalysis or scoping review
S40	S38 OR S39
S41	S36 AND S37 AND S40

Web of Science - January 6, 2021<br>Indexes=SCI-EXPANDED, SSCI, A&HCI, CPCI-S, CPCI-SSH, BKCI-S, BKCI-SSH, ESCI	
1	TOPIC:("personality disorder*")
2	TOPIC:((paranoid or schizoid or schizotypal or antisocial) NEAR/2 personalit*)
3	TOPIC:((asocial or dissocial or borderline or histrionic) NEAR/2 personalit*)
4	TOPIC:((dependent or "obsessive compulsive") NEAR/2 personalit*)
5	TOPIC:((narcissistic OR avoidant) near/2 personalit*)
6	TOPIC:(anti-social near/2 personalit*)
7	TOPIC:((passive-aggressive or passive-depressive) NEAR/2 personalit*)
8	TOPIC: ((hysterical or compulsive or inadequate) NEAR/2 personalit*)
9	TOPIC: ((explosive or asthenic or sadis* or masochis*) NEAR/2 personalit*)
10	TOPIC: ((self-defeating or negativistic or depressive) NEAR/2 personalit*)
11	TOPIC:("cluster A" and personality)
12	TOPIC:("cluster B" and personality)
13	TOPIC:("cluster C" and personality)
14	TOPIC: (BPD not (biparietal diameter or bronchopulmonary dysplasia))
15	TOPIC: (sociopath*)
16	TOPIC: (psychopath or psychopaths or psychopathy or psychopathic)
17	TOPIC: (hysteria)
18	TOPIC: (("low income" or "lower income" or "middle income") near/3 country)
19	TOPIC: (("low income" or "lower income" or "middle income") near/3 countries)
20	TOPIC: (("low income" or "lower income" or "middle income") near/3 nation)
21	TOPIC: (("low income" or "lower income" or "middle income") near/3 nations)
22	TOPIC: (("low income" or "lower income" or "middle income") near/3 econom*)
23	TOPIC: (emerging near/3 (country or countries or nation))
24	TOPIC: (emerging near/3 (nations or econom*)
25	TOPIC: (developing world)
26	TOPIC: ("third world" or "second world")
27	TOPIC: ("global south")
28	TOPIC: (LMIC*)

29	TOPIC: (Middle East* or mideast* or mid-east* or Africa or Central America* or South America* or South* Asia* or Southeast* Asia* or South east* asia* or Afghanistan or Albania* or Algeria* or American Samoa* or Angola* or Argentin* or Armenia* or Azerbaijan* or Azeri or Bangladesh* or Belarus* or Belize* or Benin* or Bhutan* or Bolivia* or Bosnia* or Herzegovin* or Botswana* or Motswana* or Brazil* or Bulgaria* or Burkina Faso or Burkinabe or Burundi* or Cape Verde* or Cabo Verde* or Cambodia* or Cameroon* or Central African Republic or Central African or Chad or Chadian or China or Chinese or Colombia* or Comoros or Comorian or Congo* or Costa Rica* or "Cote D'ivoire" or Ivorian or Cuba* or Djibouti* or Dominica* or Dominican Republic or Ecuador* or Egypt* or El Salvador* or Equatorial Guinea or Equatoguinean or Eritrea* or Eswatini or Emaswati or Ethiopia* or Fiji* or Gabon* or Gabonaise or Gambia* or Georgia* or Ghana* or Grenad* or Guatemala* or Guinea or Guinea Bissau or Bissau-Guinean or Guyan* or Haiti* or Hondura* or India* or Indonesia* or Iran* or Iraq* or Jamaica* or Jordan* or Kazakhstan* or Kenya* or Kiribati or Korea* or Kosov* or Kyrgyz* or Lao or Laos or Laotian or Lebanon or Lebanese or Lesotho or Mosotho or Basotho or Liberia* or Libya* or Madagascar or Malagasy or Malawi* or Malaysia* or Maldiv* or Mali or Malian or Marshall Islands or Marshallese or Mauritania* or Mexic* or Micronesia* or Moldova* or Mongolia* or Montenegr* or Morocc* or Mozambi* or Myanma* or Burmese or Namibia* or Nepal* or Nicaragua* or Niger* or Nigeria* or Macedonia* or Pakistan* or Papua New Guinea* or Paraguay* or Peru* or Philippines or Filipin* or Russian Federation or Russia* or Rwand* or Samoa* or Sao Tome* or Santomean or Principe or Senegal* or Serbia* or Sierra Leone* or Solomon Island* or Somali* or South Africa* or South Sudan* or Sri Lanka* or St* Lucia* or Saint Lucia* or St* Vincent or Saint Vincent or Vincentian* or Swaziland or Grenadin* or Sudan* or Surinam* or Syria* or Tajikistan* or Tanzania* or Thai* or Timor* or Togo or Togolese or Tonga* or Tunisia* or Turk* or Turkmenistan or Tuvalu* or Uganda* or Ukrain* or Uzbek* or Vanuatu* or Venezuela* or Vietnam* or West Bank or Gaza* or palestin* or "occupied territor*" or Yemen* or Zambia* or Zimbabwe*)
30	#17 OR #16 OR #15 OR #14 OR #13 OR #12 OR #11 OR #10 OR #9 OR #8 OR #7 OR #6 OR #5 OR #4 OR #3 OR #2 OR #1
31	#29 OR #28 OR #27 OR #26 OR #25 OR #24 OR #23 OR #22 OR #21 OR #20 OR #19 OR #18
32	#31 AND #30
33	TOPIC: (randomised OR randomized OR randomisation OR randomisation OR placebo*)
34	TOPIC: (random* AND (allocat* OR assign*)
35	TOPIC: (blind* AND (single OR double OR treble OR triple)
36	TOPIC: ("controlled clinical trial" or "controlled trial")
37	TOPIC: ("case report")
38	TOPIC: (editorial or comment* or "letter to the editor*")
39	TOPIC: ("systematic review" or "scoping review" or "review of reviews")

40	TOPIC: (meta-analy* or metaanaly*)
41	#40 OR #39 OR #38 OR #37 OR #36 OR #35 OR #34 OR #33

Cochrane Database: EBM Reviews - Cochrane Central Register of Controlled Trials <January 2021>, EBM Reviews - Cochrane Database of Systematic Reviews <2005 to January 6, 2021>	
1	personality disorder*.ti,ab,hw.
2	((paranoid or schizoid or schizotypal or antisocial or anti-social or asocial or dissocial or borderline or histrionic or narcissistic or avoidant or dependent or obsessive compulsive) adj2 personalit*).ti,ab,hw.
3	((passive-aggressive or passive-depressive or hysterical or compulsive or inadequate or explosive or asthenic or sadis* or masochis* or self-defeating or negativistic or depressive) adj2 personalit*).ti,ab,hw.
4	(personality and "cluster A").ti,ab,hw.
5	"cluster B".ti,ab,hw.
6	"cluster C".ti,ab,hw.
7	(BPD not (biparietal diameter or bronchopulmonary dysplasia)).ti,ab,hw.
8	sociopath*.ti,ab,hw.
9	(psychopath or psychopaths or psychopathy or psychopathic).ti,ab,hw.
10	hysteria.ti,ab,hw.
11	((low* income or middle income or developing or emerging) adj3 (country or countries or nation or nations or econom*)).ti,ab,hw.
12	developing world.ti,ab,hw.
13	(third world or second world).ti,ab,hw.
14	global south.ti,ab,hw.
15	LMIC*.ti,ab,hw.
16	((Middle East* or mideast* or mid-east* or Africa or Central America* or South America* or South* Asia* or Southeast* Asia* or South east* asia* or Afghanistan or Albania* or Algeria* or American Samoa* or Angola* or Argentin* or Armenia* or Azerbaijan* or Azeri or Bangladesh* or Belarus* or Belize* or Benin* or Bhutan* or Bolivia* or Bosnia* or Herzegovin* or Botswana* or Motswana* or Brazil* or Bulgaria* or Burkina Faso or Burkinabe or Burundi* or Cape Verde* or Cabo Verde* or Cambodia* or Cameroon* or Central African Republic or Central African or Chad or Chadian or China or Chinese or Colombia* or Comoros or Comorian or Congo* or Costa Rica* or "Cote D'ivoire" or Ivorian or Cuba* or Djibouti* or Dominica* or Dominican Republic or Ecuador* or Egypt* or El Salvador* or Equatorial Guinea or Equatoguinean or Eritrea* or Eswatini or Emaswati or Ethiopia* or Fiji* or Gabon* or Gabonaise or Gambia* or Georgia* or Ghana* or Grenad* or Guatemala* or Guinea or Guinea Bissau or Bissau-Guinean or Guyan* or Haiti* or Hondura* or India* or Indonesia* or Iran* or Iraq* or Jamaica* or Jordan* or Kazakhstan* or Kenya* or

	Kiribati or Korea* or Kosov* or Kyrgyz* or Lao or Laos or Laotian or Lebanon or Lebanese or Lesotho or Mosotho or Basotho or Liberia* or Libya* or Madagascar or Malagasy or Malawi* or Malaysia* or Maldiv* or Mali or Malian or Marshall Islands or Marshallese or Mauritania* or Mexic* or Micronesia* or Moldova* or Mongolia* or Montenegr* or Morocc* or Mozambi* or Myanma* or Burmese or Namibia* or Nepal* or Nicaragua* or Niger* or Nigeria* or Macedonia* or Pakistan* or Papua New Guinea* or Paraguay* or Peru* or Philippines or Filipin* or Russian Federation or Russia* or Rwand* or Samoa* or Sao Tome* or Santomean or Principe or Senegal* or Serbia* or Sierra Leone* or Solomon Island* or Somali* or South Africa* or South Sudan* or Sri Lanka* or St* Lucia* or Saint Lucia* or St* Vincent or Saint Vincent or Vincentian* or Swaziland or Grenadin* or Sudan* or Surinam* or Syria* or Tajikistan* or Tanzania* or Thai* or Timor* or Togo or Togolese or Tonga* or Tunisia* or Turk* or Turkmenistan or Tuvalu* or Uganda* or Ukrain* or Uzbek* or Vanuatu* or Venezuela* or Vietnam* or West Bank or Gaza* or palestin* or "occupied territor*" or Yemen* or Zambia* or Zimbabwe*) not american indian*).ti,ab,hw,hw,jn.
17	or/1-10
18	or/11-16
19	17 and 18
20	remove duplicates from 19
